# The Myelin Water Fraction Serves as a Marker for Age-Related Myelin Alterations in the Cerebral White Matter – A Multiparametric MRI Aging Study

**DOI:** 10.3389/fnins.2020.00136

**Published:** 2020-02-24

**Authors:** Tobias D. Faizy, Christian Thaler, Gabriel Broocks, Fabian Flottmann, Hannes Leischner, Helge Kniep, Jawed Nawabi, Gerhard Schön, Jan-Patrick Stellmann, André Kemmling, Ravinder Reddy, Jeremy J. Heit, Jens Fiehler, Dushyant Kumar, Uta Hanning

**Affiliations:** ^1^Department of Diagnostic and Interventional Neuroradiology, University Medical Center Hamburg-Eppendorf, Hamburg, Germany; ^2^Institute of Applied Biometrics and Epidemiology, University Medical Center Hamburg-Eppendorf, Hamburg, Germany; ^3^Institute of Neuroimmunology and Multiple Sclerosis, University Medical Center Hamburg-Eppendorf, Hamburg, Germany; ^4^Department of Neurology, University Medical Center Hamburg-Eppendorf, Hamburg, Germany; ^5^Department of Diagnostic and Interventional Neuroradiology, University Medical Center Muenster, Münster, Germany; ^6^Department of Radiology, University of Pennsylvania, Philadelphia, PA, United States; ^7^Department of Radiology, Stanford University School of Medicine, Palo Alto, CA, United States

**Keywords:** T2-relaxometry, myelin water imaging, diffusion imaging, magnetization transfer imaging, normal aging

## Abstract

Quantitative MRI modalities, such as diffusion tensor imaging (DTI) or magnetization transfer imaging (MTI) are sensitive to the neuronal effects of aging of the cerebral white matter (WM), but lack the specificity for myelin content. Myelin water imaging (MWI) is highly specific for myelin and may be more sensitive for the detection of changes in myelin content inside the cerebral WM microstructure. In this multiparametric imaging study, we evaluated the performance of myelin water fraction (MWF) estimates as a marker for myelin alterations during normal-aging. Multiparametric MRI data derived from DTI, MTI and a novel, recently-proposed MWF-map processing and reconstruction algorithm were acquired from 54 healthy subjects (aged 18–79 years) and region-based multivariate regression analysis was performed. MWFs significantly decreased with age in most WM regions (except corticospinal tract) and changes of MWFs were associated with changes of radial diffusivity, indicating either substantial alterations or preservation of myelin content in these regions. Decreases of fractional anisotropy and magnetization transfer ratio were associated with lower MWFs in commissural fiber tracts only. Mean diffusivity had no regional effects on MWF. We conclude that MWF estimates are sensitive for the assessment of age-related myelin alterations in the cerebral WM of normal-aging brains.

## Introduction

Myelin holds a crucial role in the composition of brain’s microstructure and is an important compartment of the cerebral white matter (WM). During normal-aging, structural components within the human brains’ white- and gray matter undergo extensive biophysiological changes (Pannese, 2011). Brain maturation, as well as regressive processes affecting myelin integrity occur in a time-displaced manner throughout different brain regions (Haroutunian et al., 2014). Evidence suggests that protracted myelination continues from early infanthood to the fourth decade of life (Peters, 2002; Billiet et al., 2015), while extensive age-related myelin alterations occur during late adulthood up until old age (Gunning-Dixon et al., 2009; Callaghan et al., 2014; Bennett et al., 2017). Histopathological studies reported that normal brain aging is marked by degeneration of WM microstructure including myelin pallor (Kemper, 1994), loss of myelinated nerve fibres (Bartzokis, 2004; Bartzokis et al., 2004), malformation of myelin sheaths (Peters, 2002; Gunning-Dixon et al., 2009), as well as disrupted macrostructural organization concomitant with an enlargement of the extracellular space (Salat et al., 2005; Bennett et al., 2010; Billiet et al., 2015). Therefore, an accurate *in vivo* assessment of myelin content is both, instructive and important (MacKay and Laule, 2016). Conventional magnetic resonance imaging (MRI) provides an excellent spatial depiction of the brain, but lacks specificity for the evaluation of microstructural brain changes (MacKay and Laule, 2016). In contrast, quantitative MRI techniques such as diffusion tensor imaging (DTI) or magnetization transfer imaging (MTI) have shown to be more specific to age-related changes of the cerebral WM microstructure. Imaging markers derived from diffusion-based MRI such as fractional anisotropy (FA), radial diffusivity (RD), or mean diffusivity (MD) have been reported as general indicators of microstructural status, since they are sensitive to alterations in the cell density, orientation of cell membranes, as well as cell sizes or numbers (Billiet et al., 2015) and thus only indirectly to myelin. Alterations of diffusion parameters with age have been reported in several previous studies (Salat et al., 2005; Bennett et al., 2010; Lebel et al., 2012; Billiet et al., 2015; Jang and Seo, 2015). However, diffusion-based measurements cannot directly assess myelin content (Billiet et al., 2015). MTI studies have reported potential evidence for age-related demyelination (Mehta et al., 1995; Pike et al., 2000; Rovaris et al., 2003; Gunning-Dixon et al., 2009), albeit MTI measures also lack specificity for myelin (Serres et al., 2009; Vavasour et al., 2011) and need confirmation using techniques more sensitive to myelin content (Billiet et al., 2015). Myelin water imaging (MWI), using multi-exponential T2 (MET2) data, shows superior sensitivity for myelin content (Billiet et al., 2015; Faizy et al., 2016; MacKay and Laule, 2016) and the myelin water fraction (MWF) has been established to be a potential *in vivo* marker for myelin content (MacKay and Laule, 2016). However, MET2 studies elucidating age-dependant alterations of cerebral WM patterns are scarce (Lang et al., 2014; Billiet et al., 2015; Arshad et al., 2016) and limited information exists about the development of MWF metrics in relationship with other quantitative MRI approaches during the process of aging (Billiet et al., 2015). Moreover, new and improved MWI approaches (enabling whole brain coverage in clinically feasible acquisition time) have been developed in the recent past, which need to be validated and tested in the clinical routine outside of a research environment. In this investigation, we have employed a recently proposed iterative multi-voxel spatially regularized MWI reconstruction approach (Kumar et al., 2018), which enabled enhanced noise robustness of reconstruction along with a more accurate accounting for the stimulated echo contribution. Consequently, this resulted in considerably improved MWF-quantifications, especially in the sub-cortical- and major WM tract regions.

The purpose of this multimodal imaging study was to evaluate the sensitivity of MWF estimates as a marker for age-dependent myelin alterations in major WM regions, utilizing a recently proposed MWF-map reconstruction approach (Kumar et al., 2018). We sought to investigate region-based relationships of MWF estimates with other, well-established biomarkers sensitive for microstructural WM changes, derived from magnetization-based imaging [magnetization transfer ratio (MTR) and DTI (FA, MD, RD)]. We hypothesized that the improved noise robustness of reconstruction and the more accurate accounting of stimulated echo contributions of our MWI processing method would lead to an enhanced sensitivity of MWF measures to age-related myelin alterations within the cerebral microstructure. We presumed a decrease of mean MWF measures from mid-adulthood to old-age throughout several WM regions of interest (ROI), while expecting myelination to be preserved during younger life decades and in brain regions that consist of fibers derived from primary motor- or sensory pathways.

## Materials and Methods

### Subjects’ Characteristics

**FIGURE 1:**
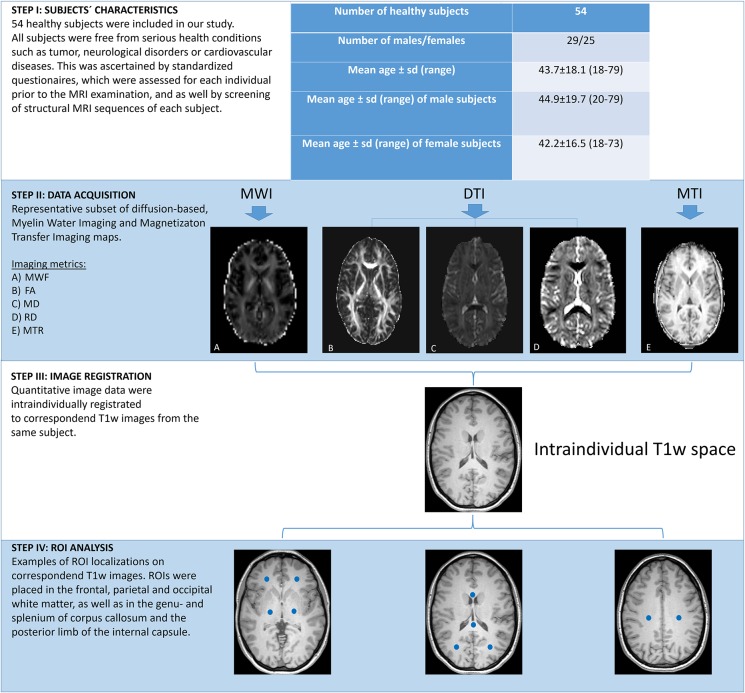
Work-flow of post-acquisition processing steps. Figure presents subjects’ characteristics and visualizes distinct steps in the work-flow of image processing.

Fifty four healthy subjects were enrolled in our study (25 males, 29 females). Median age was 43 years (age range 18–79 years). Patient characteristics are displayed in the work-flow chart Figure 1. The study was approved by the local research Ethical Committee Hamburg (Ethik-Komission der Ärztekammer Hamburg) following the guidelines of the Declaration of Helsinki and written informed consent was obtained from each subject. All individuals were known to be free of any physical and mental diseases. This was ascertained by standardized questionnaires, which included several queries ‘concerning the subjects’ general health condition, as well as questions that evaluated the subjects’ former health history. All forms were reviewed by the supervising physician before any MRI scan was performed. Structural MRI sequences, 3D MPRAGE images and fluid attenuated inversion recovery sequences (FLAIR), were surveyed to exclude pathological brain processes (e.g., tumors, post-ischemic/post-hemorrhage defects, etc.). Leukoaraiosis was considered as a regular process of aging but only subjects with a Fazekas score (Fazekas et al., 1987) of ≤ 1 were included in our study to prevent possible compromise of the measured data.

### MRI Data Acquisition

All MRI scans were conducted on a 3T scanner (Ingenia, Philips Healthcare, Best, Netherlands) with a 32-channel head coil. The protocol consisted of axial 3D FLAIR images: TR = 4800 ms, TE = 289 ms, TI = 1650, Matrix: 224 × 224 × 192, voxel size = 0.5 mm × 0.5 mm × 1.1 mm, ETL = 167; sagittal 3D MPRAGE with TR = 6.4 ms, TE = 2.9 ms, matrix = 256 × 256 × 192, voxel size = 0.94 mm × 0.94 mm × 0.94 mm, ETL = 256, TI = 1120 ms, excitation RF flip angle 6°. 3D non-selective GRASE sequence was acquired with 3 mm isotropic voxel size and the following parameters: echo spacing = 6 ms; number of echoes = 32 (maximum TE = 193 ms; TR = 2000 ms), FOV = 240 mm × 192 mm; ETL = 96; number of slices = 64 slices; Parallel-imaging = SENSE; SENSE factor = 1.5 was used in both phase encoding directions; Foldover Suppression = No; Over-contiguous slices = No; Number of averages = 1; slice oversampling factor = 1; sagittal orientation; acquisition time: 15 min. Sagittal slice orientation was used for acquisition to avoid folding-in artifacts in head-foot direction, since non-selective RF-pulses were used. 3D-MTR maps were acquired using a pair of T1w gradient recalled echo volumes with TR = 69 ms, TE = 2.2 ms, 192 slices, slice thickness = 2.0 mm, matrix = 120 × 120, voxel size = 1.8 mm × 1.8 mm × 2.0 mm, ETL = 1, flip angle = 10°, SENSE factor = 2 with/without a Gaussian magnetization transfer contrast (MTC) prepulse (MTCon or MTCoff) with 180 Hz bandwidth, 1.2 kHz offset frequency, 10 ms duration and 500° flip angle. Axial single-shot echo planar DTI sequence was obtained using 20 non-colinear diffusion-weighted gradient directions with TR = 3800 ms, TE = 74 ms, slice thickness = 5 mm, interpolated matrix = 256 × 256, voxel size = 0.9 mm × 0.9 mm × 5 mm, FOV = 230 × 230 mm, ETL = 45, SENSE factor = 2, gradient b value 0, 1000 s/mm^2^. DTI raw data were corrected for eddy current and motion before the diffusion tensor was fitted for each brain voxel.

### Data Processing

#### Calculation of MWF Maps, DTI Scalars and MTR

Myelin water fraction maps were extracted from 3D multi echo GRASE data using a recently proposed alternate-minimization multi-voxel spatial regularization (MVSR) approach (Kumar et al., 2018). MTR maps and DTI scalars were calculated using tools included in the Oxford Centre for Functional MRI of the Brain Software Library (FSL, FMRIB Analysis Group, University of Oxford, Oxford, United Kingdom). Magnetization Transfer Ratio (MTR) values were calculated using following formula: MTR = (MTC_OFF_ – MTC_ON_)/MTC_ON_.

#### Regions of Interest Analysis and Image Registration

All image data were processed using the software package FSL 5.0 (Analysis Group, FMRIB, Oxford, United Kingdom). For image registration, the software packages R (version 3.3.4) and R studio (version 3.4.0) including the package extrantsR^1^ were utilized. Preprocessing of the acquired image data was performed with in-house scripts. Regions of Interest based analysis was performed with the software package ANALYZE 11.0 (Analyze Direct, Overland Park, KS, United States). For image registration, corresponding DTI and MTI images, as well as the MWF maps of each subject were intraindividually registered to the corresponding MPRAGE images of each subject with a series of linear and non-linear (affine) registrations. Registration quality was assessed by an experienced neuroradiologist (T.D.F.). In each subject, a total number of 10 ROIs were defined in distinct brain regions on each corresponding MPRAGE images. ROIs of 6 mm^3^ oval size were outlined in the frontal, parietal and occipital WM (including the optic radiation) and in the corticospinal tracts (CST, posterior limb of the internal capsule) on both hemispheres and also in the genu- (GCC) and splenium (SCC) of corpus callosum. Binary ROI masks were saved. Subsequently, the inverse transformation matrices were used to map the ROIs to the DTI and MTI and MWI spaces. Finally, the ROIs were multiplied with the corresponding MTR, MWF and FA/MD/RD maps and region-based measurements were obtained for each subject. Figure 1 exemplifies the work-flow of the post-acquisition processing steps.

### Statistical Analysis

Data are presented as mean ± standard deviation (SD). Normal distribution of the data was tested with the Kolomogorov–Smirnov test. Region-based associations of the acquired MRI parameters (MWF, FA, RD, and MTR) with age were tested using Pearson’s correlation coefficient on a linear basis. The unit of RD and MD is (x10^–3^ mm^2/^s). Paired two-tailed *t*-tests were computed to test for differences of mean MWFs between the brain hemispheres of each subject.

Additionally, we performed a multivariate regression analyses to investigate the association of regional MWF calculations with age, diffusion tensor-, as well as magnetization transfer measures. In each multivariate regression analysis, MWF was the dependent variable in different ROIs. To improve the interpretability, parameter scales were adjusted: all MRI biomarkers are displayed multiplied by factor 10, while age was divided by 10. The effect plots show the model-based effects of each independent variable – under control of all variables in the model – on the outcome; for example, frontal MWF (as displayed in Figure 3). For the variable “age,” the respective effect models’ correlation coefficients are estimated using age in decades. In addition, we also show the bivariate relationships between the MWF and the other distinct MRI parameters and age in the effect plots (light gray colored plots) and also plot the datapoints, the regression lines, the confidence intervals (CIs) and *p*-values. Statistical analyses were performed in SPSS version 24 (IBM Corporation, Armonk, NY, United States) and R: A Language and Environment for Statistical Computing (R Foundation for Statistical Computing, Vienna, Austria, 2019).

## Results

### Differences of the MRI Parameters Between the Left and Right Hemisphere

The mean MWF, FA, MD, RD, and MTR values obtained from both hemispheres were comparable and no significant side-differences were found between the respective ROIs in the frontal, parietal, and occipital WM and CST of both sides (*p* > 0.05 for all ROIs). Hence, since no significant side-differences were detected, mean measurements of bilateral structures were combined into single regions and we referred to these measures as frontal WM, parietal WM, occipital WM and CST. The distribution of all MRI parameter measures throughout the age decades is displayed in Supplementary Figures 1a–e.

### Progression of MRI Parameter With Age

**FIGURE 2:**
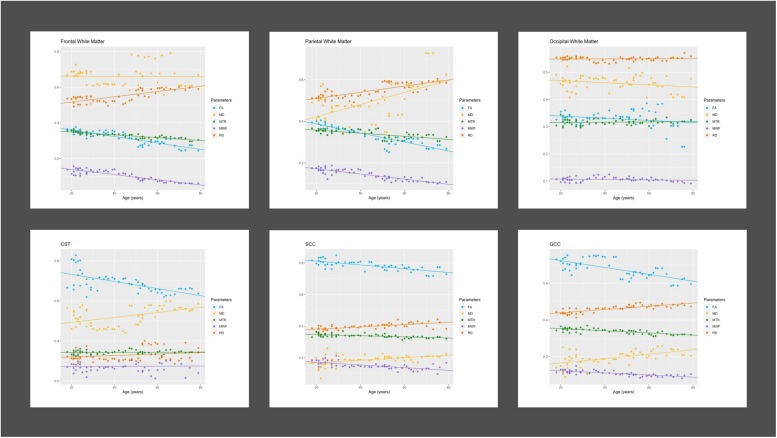
Progression of MRI parameters with age in different brain regions. Figure displays age-related regional progressions of Diffusion Tensor scalars [fractional anisotropy (FA); mean diffusivity (MD); radial diffusivity (RD); magnetization transfer ratio (MTR); myelin water fraction (MWF)]. CST, corticospinal tract; GCC, genu of corpus callosum; SCC, splenium of corpus callosum.

Figure 2 displays the progression of the mean values of all derived MRI parameter with age grouped by ROI localizations. Table 1 summarizes the results of bivariate correlations between the distinct MRI parameter and age. No significant differences between male and female participants were found for the here presented trends. MWF, FA, RD, and MTR showed moderate to strong significant correlations with age in the frontal and parietal WM ROIs, as well as in the GCC and SCC. Only FA and MD showed significant correlations with age in CST ROIs. Only MWF showed a significant but low negative correlation with age in occipital WM ROIs.

**TABLE 1:** Bivariate correlations of the different MRI parameters with age.

Region of interest	MWF	FA	MD	RD	MTR
Frontal WM	−0.87**	−0.83**	0.55**	0.73**	−0.85**
Parietal WM	−0.86**	−0.88**	0.67**	0.81**	−0.70**
Occipital WM	−0.22*	–0.14	–0.17	–0.25	0.1
GCC	−0.64**	−0.76**	0.55**	0.75**	−0.55*
SCC	−0.62**	−0.74**	0.45**	0.64**	−0.7**
CST	0.41	−0.64**	0.44**	0.12	0.22

Values in the columns display Pearson’s r in relation to subjects’ age. Asterisks indicate the level of significance (*p ≤ 0.05; **p < 0.001). WM, white matter; GCC, genu of corpus callosum; SCC, splenium of corpus callosum; CST, corticospinal tract; MWF, myelin water fraction; FA, fractional anisotropy; MD, mean diffusivity; MTR, magnetization transfer ratio.

### Region-Based Age Dependence of MWF Measures, Diffusion-Based Metrics and Magnetization Transfer Contrast

**FIGURE 3:**
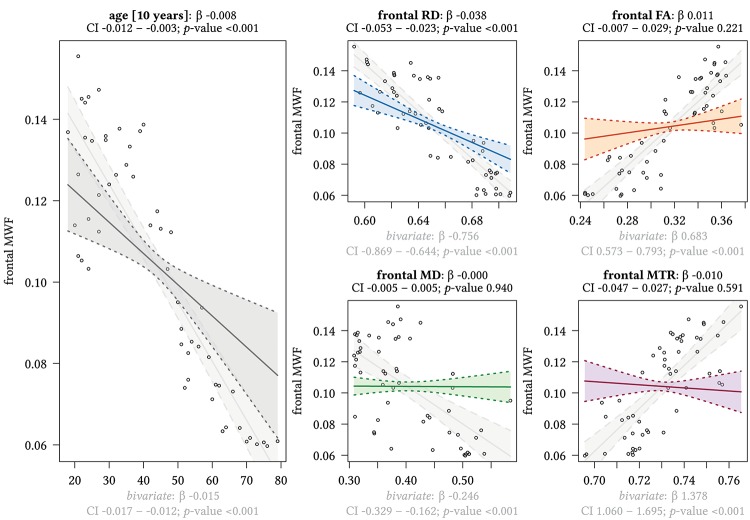
Region-based relationships of MWF metrics with diffusion-based scalars, MTR and age in the frontal WM. The Effect plots in figure show the marginal effects of the multivariate regression model. For each of the five independent variables [age: gray plot; Radial Diffusivity: blue plot: Fractional Anisotropy: orange plot: Mean Diffusivity: green plot: Magnetization Transfer Ratio: purple plot] we show the model-based effect on the MWF measured in the frontal white matter. The regression coefficient (β), confidence interval (CI, 2.5–97.5%) and *p*-value for the multivariate model are shown above the respective plot region. For the variable “age,” the model’s regression coefficient is estimated using age in decades. Additionally, we show the bivariate unadjusted correlation (points, regression line, and CI in light gray in the background). The respective regression coefficient for the bivariate model, the related CI and *p*-value are shown below the respective plot region in light gray.

**FIGURE 4:**
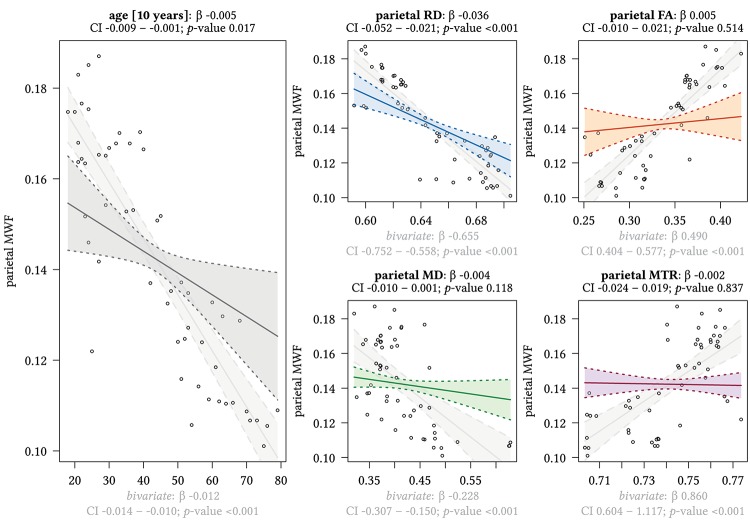
Region-based relationships of MWF metrics with diffusion-based scalars, MTR and age in the parietal WM. The Effect plots in figure show the marginal effects of the multivariate regression model. For each of the five independent variables [age: gray plot; Radial Diffusivity: blue plot: Fractional Anisotropy: orange plot: Mean Diffusivity: green plot: Magnetization Transfer Ratio: purple plot] we show the model-based effect on the MWF measured in the parietal white matter. The regression coefficient (β), confidence interval (CI, 2.5–97.5%) and *p*-value for the multivariate model are shown above the respective plot region. For the variable “age,” the model’s regression coefficient is estimated using age in decades. Additionally, we show the bivariate unadjusted correlation (points, regression line, and CI in light gray in the background). The respective regression coefficient for the bivariate model, the related CI and *p*-value are shown below the respective plot region in light gray.

**FIGURE 5:**
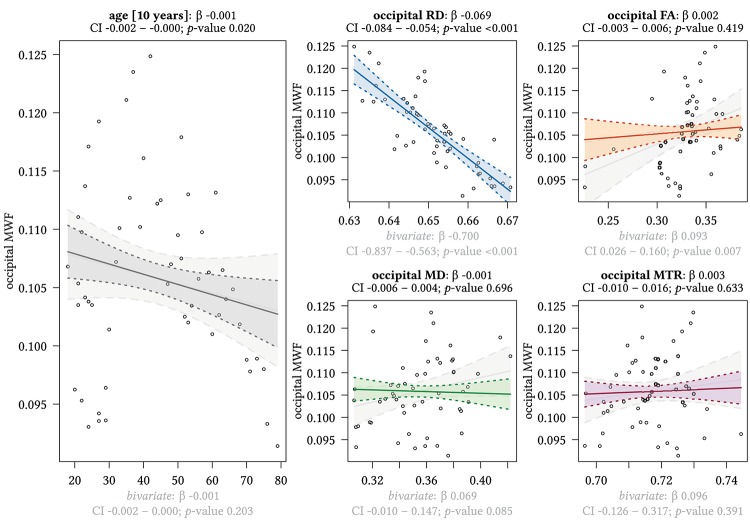
Region-based relationships of MWF metrics with diffusion-based scalars, MTR and age in the occipital WM. The Effect plots in figure show the marginal effects of the multivariate regression model. For each of the five independent variables [age: gray plot; Radial Diffusivity: blue plot: Fractional Anisotropy: orange plot: Mean Diffusivity: green plot: Magnetization Transfer Ratio: purple plot] we show the model-based effect on the MWF measured in the occipital white matter. The regression coefficient (β), confidence interval (CI, 2.5–97.5%) and *p*-value for the multivariate model are shown above the respective plot region. For the variable “age,” the model’s regression coefficient is estimated using age in decades. Additionally, we show the bivariate unadjusted correlation (points, regression line, and CI in light gray in the background). The respective regression coefficient for the bivariate model, the related CI and *p*-value are shown below the respective plot region in light gray.

**FIGURE 6:**
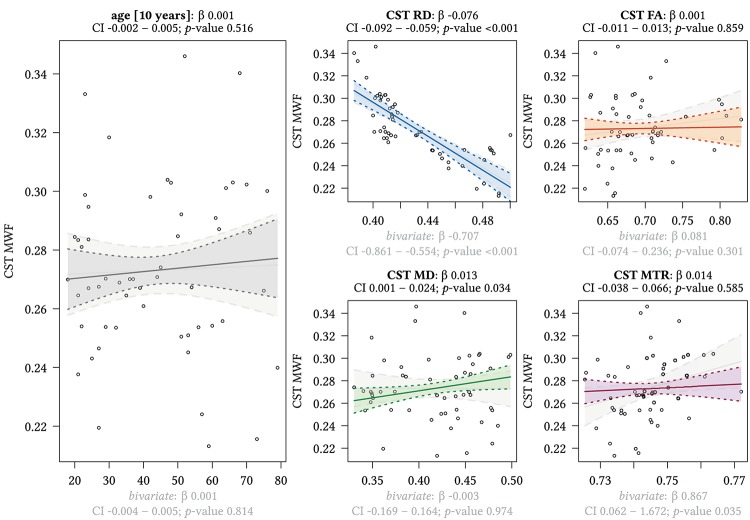
Region-based relationships of MWF metrics with diffusion-based scalars, MTR and age in the CST. The Effect plots in figure show the marginal effects of the multivariate regression model. For each of the five independent variables [age: gray plot; Radial Diffusivity: blue plot: Fractional Anisotropy: orange plot: Mean Diffusivity: green plot: Magnetization Transfer Ratio: purple plot] we show the model-based effect on the MWF measured in the corticospinal tract (CST). The regression coefficient (β), confidence interval (CI, 2.5–97.5%) and *p*-value for the multivariate model are shown above the respective plot region. For the variable “age,” the model’s regression coefficient is estimated using age in decades. Additionally, we show the bivariate unadjusted correlation (points, regression line, and CI in light gray in the background). The respective regression coefficient for the bivariate model, the related CI and *p*-value are shown below the respective plot region in light gray.

**FIGURE 7:**
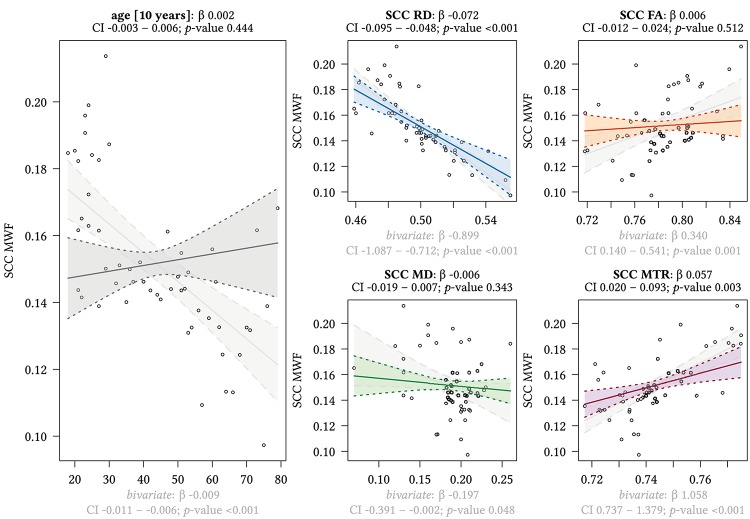
Region-based relationships of MWF metrics with diffusion-based scalars, MTR and age in the SCC. The Effect plots in figure show the marginal effects of the multivariate regression model. For each of the five independent variables [age: gray plot; Radial Diffusivity: blue plot: Fractional Anisotropy: orange plot: Mean Diffusivity: green plot: Magnetization Transfer Ratio: purple plot] we show the model-based effect on the MWF measured in the splenium of the corpus callosum (SCC). The regression coefficient (β), confidence interval (CI, 2.5–97.5%) and *p*-value for the multivariate model are shown above the respective plot region. For the variable “age,” the model’s regression coefficient is estimated using age in decades. Additionally, we show the bivariate unadjusted correlation (points, regression line, and CI in light gray in the background). The respective regression coefficient for the bivariate model, the related CI and *p*-value are shown below the respective plot region in light gray.

**FIGURE 8:**
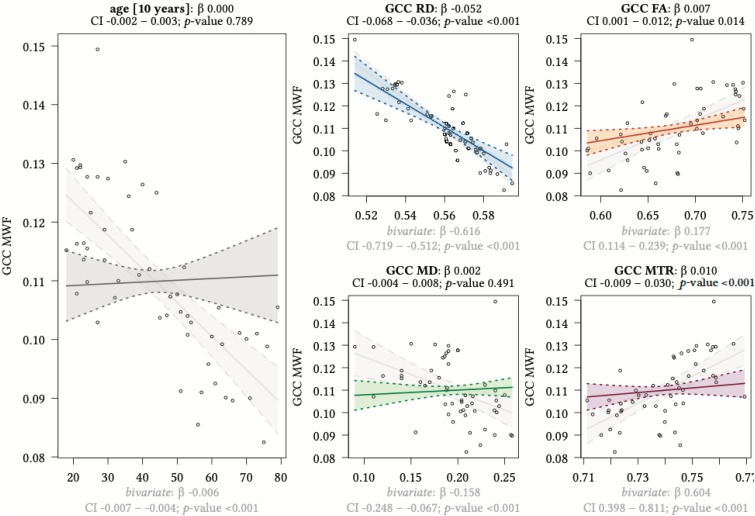
Region-based relationships of MWF metrics with diffusion-based scalars, MTR and age in the GCC. The Effect plots in figure show the marginal effects of the multivariate regression model. For each of the five independent variables [age: gray plot; Radial Diffusivity: blue plot: Fractional Anisotropy: orange plot: Mean Diffusivity: green plot: Magnetization Transfer Ratio: purple plot] we show the model-based effect on the MWF measured in the genu of the corpus callosum (GCC). The regression coefficient (β), confidence interval (CI, 2.5–97.5%) and *p*-value for the multivariate model are shown above the respective plot region. For the variable “age,” the model’s regression coefficient is estimated using age in decades. Additionally, we show the bivariate unadjusted correlation (points, regression line and CI in light gray in the background). The respective regression coefficient for the bivariate model, the related CI and *p*-value are shown below the respective plot region in light gray.

Figures 3–8 and Supplementary Table 1 display the results of multivariate regression analysis with MWF as the dependent variable in different ROIs and age as the independent variable. In addition, Figures 3–8 also show the results for bivariate correlations between the distinct parameters for comparison. Subjects’ sex had no significant influence on the effect of MWF on either age or the other distinct MRI parameters in any brain region investigated.

We detected a significant relationship between MWF and RD metrics in all investigated WM ROIs (*p* < 0.001 for all ROIs) under control of all variables included in the regression model. Moreover, a significant effect of age on MWF was found for frontal (*p* < 0.001), parietal (*p* = 0.017), and occipital (*p* = 0.02) WM ROIs. Interestingly, controlled against all other variables in the model, no significant association of MWF and age was found for the GCC (*p* = 0.789) and SCC (0.44) in multiparametric regression analysis. Bivariate correlations of MWF and age in Figure 2 clearly indicate a reduction of mean MWF measures in the GCC and SCC with age. Moreover, a mild relationship between MD and MWF was found for the CST (*p* = 0.034). We detected an association of MTR with MWF in the SCC (*p* = 0.003) and GCC (*p* < 0.001).

## Discussion

The purpose of this study was to evaluate the performance of MWF estimates as a marker for age-dependent myelin alterations in the cerebral WM and to evaluate the association of MWF estimates with other MRI biomarkers indicating age-related microstructural changes, derived from DTI and MTI. We hypothesized that MWF metrics were sensitive for the detection of regional- and age-dependent alterations of myelin content in the cerebral WM pattern throughout the lifespan, reflecting a portion of the ongoing processes of re- and demyelination that physiologically occur in normal-aging brains. The obtained MWF values decreased with age and showed different degrees of regional associations with other imaging biomarkers associated with age-dependent WM changes, hence confirming previous assumptions about age-dependent alterations in the cerebral WM of healthy individuals (Billiet et al., 2015; Arshad et al., 2016).

### Age-Related Decreases of Regional MWFs May Reflect Myelin Content and Are Associated With RD, but Not With FA and MD

Pathophysiological investigations revealed that myelination status considerably changes throughout the life decades (Peters, 2002; Salat et al., 2005; Pannese, 2011; Haroutunian et al., 2014). Considerable histopathological evidence suggests that protracted myelination (and thus an equilibrium of myelination and demyelination resulting in a preservation of myelin content) is present until 4–6^*th*^ life decades, followed by a notable decrease of WM myelination toward higher age decades (Gunning-Dixon et al., 2009; Arshad et al., 2016). Accordingly, biomarkers sensitive for these specific developmental patterns of age-related myelin changes are of great interest for researchers studying aging. While many related imaging studies predominantly reported of linear declines of diffusion-based metrics (Bennett et al., 2010; Lebel et al., 2012; Billiet et al., 2015; Rathee et al., 2016), only very few studies found a correlation of MWF estimates with age (Billiet et al., 2015; Arshad et al., 2016; Faizy et al., 2018; Bouhrara et al., 2019). Histopathologically, normal brain-aging is marked by WM degeneration, including myelin pallor (Kemper, 1994), loss of myelinated nerve fibers (Bartzokis, 2004; Bartzokis et al., 2004) and malformation of myelin sheaths (Peters, 2002; Gunning-Dixon et al., 2009). Since the MWF is believed to be an *in vivo* proxy sensitive for myelin content (MacKay and Laule, 2016), we presumed that age-related alterations of these estimates may reflect changes of WM myelination during normal aging. Recent multimodal studies including different MWI approaches (Billiet et al., 2015; Arshad et al., 2016) have reported contradictive findings in terms of age-dependencies of MWF measures. In addition to DTI measures, Arshad et al. (2016) utilized the method developed by Prasloski et al. (2012b) and detected heterochronic associations between MWF and age in a cohort of 61 healthy subjects. Moreover, the study reported of regional inverted U-shaped progressions of MWF values with age in subcortical WM tracts and commissural fibers, while DTI measures showed no such pattern and concluded that MWF estimates may reflect myelin content during normal aging. Moreover, in their study, MWF estimates were mostly unrelated to DTI proxies throughout several WM ROIs. Billiet et al. (2015) investigated age-related patterns of microstructural brain changes with DTI, multishell diffusion MRI (dMRI) and MET2 metrics based on the MWI reconstruction approach by Prasloski et al. (2012b). The authors only found mild correlations of MWF measures with age in few WM regions (fornix crescent and stria terminalis). Moreover, they did not find any association of MWF with “conventional” DTI metrics (FA, MD, RD, AD), but reported of correlations between measures of dMRI [namely neurite orientation dispersion and density imaging (NODDI) and diffusion kurtosis imaging (DKI)] and MWF estimates and concluded that MET2 metrics are less sensitive to age-related differences during early to mid-adulthood than diffusion measures (Billiet et al., 2015). Aside from methodological differences, the diverging findings of both related studies (and ours) lead to the assumption that the existence (or absence) of correlations between DTI and MWF estimates may at least be partially attributed to the utilization of different MWI techniques (see below).

Our results are in line with previous reports of age-related decreases of FA and MTR and as well with findings of increasing RD and MD measures during normal aging (Mehta et al., 1995; Salat et al., 2005; Davis et al., 2009; Bennett et al., 2010; Lebel et al., 2012; Callaghan et al., 2014; Billiet et al., 2015; Arshad et al., 2016). As explored before, discordant findings have been reported regarding the associations of MET2 metrics and diffusion-based measures. For the greater part, these reports are in accordance with our findings, since we also detected no significant relationship of MWF with FA or MD in most ROIs. FA describes the degree of non-isotropic diffusion, while MD is the average amount of water diffusion (average diffusivity of all three Eigenvalues) (Billiet et al., 2015; Bennett et al., 2017). Both measures are commonly used in related aging studies and have shown to be sensitive for several degenerative microstructural processes ‘throughout the brain’s WM, such as changes in cell density, orientation, size and numbers, as well as volume of axonal fibers (Beaulieu, 2002). Consequently, FA and MD are not directly related to myelin content (Rovaris et al., 2003), which may explain the missing link between MWF and FA/MD. RD corresponds to diffusion in perpendicular direction of axonal fiber tracts (Bennett et al., 2010) and was reported to be more sensitive for changes of myelination status than FA/MD (Song et al., 2002). Moreover, while some recent studies did not find any relationship between diffusion-based metrics and age (Arshad et al., 2016), a predominant number of comprehensive multimodal studies reported of regional age-related quadratic associations of RD (and FA) (Salat et al., 2005; Bennett et al., 2010; Hasan et al., 2010; Bartzokis et al., 2012; Lebel et al., 2012; Billiet et al., 2015). Moreover, RD was reported to be a valid marker of myelin integrity, rather than a proxy for axonal loss or macrostructural disruption in studies including healthy individuals, Multiple Sclerosis patients and mouse models (Song et al., 2002, 2005; Wang et al., 2015). These findings may explain the association between MWF estimates and RD metrics, as detected in our study. Furthermore, associations of RD and MWF have been reported before for ROIs containing larger fiber diameters, such as CST, optic radiation (as represented by the occipital WM ROI in our study) and corpus callosum (Madler et al., 2008; Arshad et al., 2016), which is also supported by our findings. Furthermore, our findings indicate that an association between these two parameters is also present in frontal and parietal WM regions. Regarding the correlations of MTI measures with MWF, it is noteworthy that we detected an association between MWF and MTR only in the two corpus callosum ROIs. MTI based metrics indirectly measure the interaction between mobile water protons and protons residing within or intimately associated with CNS macromolecules (Smith et al., 2009) and have been reported to decrease in normal-aging brains (Mehta et al., 1995; Rovaris et al., 2003) or demyelinating diseases such as Multiple Sclerosis (Ropele and Fazekas, 2009). Reductions of MTR indicate a reduced ability of macromolecules in tissue to exchange magnetization with surrounding water molecules, primarily attributed to axonal loss and damage (Rovaris et al., 2003; Schiavone et al., 2009). Previous histopathological investigations (Pierpaoli et al., 2001; Beaulieu, 2002; Bennett et al., 2010; Hasan et al., 2010) also suggested a relationship between the density of axonal packing and several age-related microstructural processes (e.g., degree of myelination, axonal diameter, fiber density) that may influence the amount of extracellular water components in the vicinity of densely packed myelinated nerve fibers, as present in the corpus callosum. The authors in Bennett et al. (2010) concluded that severe decreases in axonal packing density, e.g., due to a greater loss of myelin or axons in aging, may result in a global increase of extracellular water. Consequently, increased amounts of (relatively free) water molecules would lead to a reduction of MTR measures, while at the same time, considerable loss of total myelin content and consecutive increases of extracellular water would also lead to decreased MWFs. Therefore, these circumstances may explain the effect of MTR estimates on MWF and may account for the simultaneous decreases of both measures in densely organized trajectories such as the corpus callosum.

However, interestingly, no significant MWF reduction was found in the CST and only minor MWF changes were detected in the occipital WM indicating that myelination in these two regions may not be affected by significant changes during normal aging. These findings are strengthened by histopathological studies reporting that brain areas, which contain fiber tracts originating from central brain regions (motor or sensory pathways, auditory and visual pathways; as partially represented by the CST and occipital WM ROI in our investigation) are well-preserved from extensive degrees of age-related myelin loss (Salat et al., 2005; Wu et al., 2011).

Noteworthy, some studies reported that in some brain regions (with emphasis on anterior brain areas), non-linear progressions of myelin proxies with age may be in closer agreements with histopathological findings of a preservation of myelin content during the first four decades of life, followed by a notable decrease of myelination in higher life decades, compared to linear trends (Peters, 2002; Lebel et al., 2012; Callaghan et al., 2014; Arshad et al., 2016). For instance, already in Flynn et al. (2003) reported of protracted myelination in healthy individuals during the first decades of life, which reflects the statistical trends that have been reported in recent MWI studies (Arshad et al., 2016; Bouhrara et al., 2019). More extensive (longitudinal) imaging studies including higher subject numbers would be desirable to further validate these claims in terms of MWF estimates throughout different brain regions.

### Technical and Methodological Considerations

Myelin water fraction reconstruction, from time series of T2 weighted data, is known to be very sensitive to signal-to-noise ratios (Graham et al., 1996) and also requires an accurate accounting for stimulated echo contributions (Kumar et al., 2018). MWF-mapping, using the recently proposed MWI reconstruction approach (Kumar et al., 2018) has been shown to result in improved noise robustness and better convergence compared to competing approaches (Prasloski et al., 2012a; Kumar et al., 2016). The resultant improved sensitivity has enabled us to detect age-related MWF changes more accurately. This may explain, why we were able to detect both, a decrease of MWF estimates with age and regional dependent associations of MWFs with other parameters related to myelin content (RD, MTR), while other studies did not find these relationships. However, current MWI approaches still may not reliably distinguish between intermediate and long T2 pools present inside the same structure/pathology, as most of MWI protocols only utilize maximum TE < 300 ms. Although TE < 300 ms is sufficient for the accurate detection of MWF values, an accurate distinction between intermediate and long T2 pools would require maximum TE ∼800 ms (Laule et al., 2007; Kumar et al., 2018). In this regard, despite being challenging, the detection of long-T2 pools may be very informative in certain pathological (demyelinating) processes (Lang et al., 2014; Alonso-Ortiz et al., 2015; MacKay and Laule, 2016; Kumar et al., 2018).

Due to the lack of histological validation, it is of enormous difficulty to compare the specific findings of all related multimodal MRI studies (including ours) to each other, since most of them utilized different analysis methods (voxel-based vs. ROI-based vs. whole brain comparisons), different scanner types, different diffusion or MTI sequences and predominantly different MWI approaches. Therefore, we presume that findings of *in vivo* multiparametric MRI studies need to be interpreted in context with histopathology and in synopsis with the underlying specific technical and methodological approaches. After all, the physiology of age-related changes inside the cerebral WM microstructure is a complex and heterogeneous process that is associated with a high degree of inter-individual variability (Gunning-Dixon et al., 2009).

### Limitations

In our study, a ROI-based investigation approach was used to evaluate age-related microstructural WM changes in distinct brain localizations. The pros and contras of region-based analysis against whole-brain assessments, such as voxel-wise or histogram analysis have been discussed before (Schiavone et al., 2009; Billiet et al., 2015). While ROI-based approaches intrinsically only cover certain brain regions and do not provide information of the whole-brain; voxel-wise analysis may lack spatial information and are prone to partial volume effects in certain brain regions such as gray-white- and CSF-WM boundaries, as well as possible confounds by Leukaraiosis and other structural abnormalities. However, ROI-based approaches are susceptible to subjective bias and derived values may vary, if different ROI sizes are utilized due to partial volume effects or erroneous measurement of adjacent brain structures. In the absence of the histopathological “gold-standard” verification, the decrease in MWF cannot be attributed to demyelination only. In fact, several competing pathophysiological processes, such as iron deposition in the WM, loss of myelinated nerve fibers, malformation of myelin sheaths and myelin pallor can be contributing factors as well (Pannese, 2011; Sala et al., 2012; Liu et al., 2017; Birkl et al., 2019). While our sample size, consisting of 54 subjects covering a lifespan of more than 60 years is comparable to other related imaging studies (Billiet et al., 2015; Arshad et al., 2016), it may not be sufficiently big to draw clinically valid inferences. However, since the aim of this investigation was to evaluate age-related associations of MWF metrics with other MRI parameters within the same subject, we believe that the number of subjects included is sufficient to explore the associations between MWF, DTI, and MTI estimates in different ages of life. We utilized a 3D GRASE MWI sequence with a TE maximum of 193 ms. While Kumar et al. 2018; (Supplementary Figure 1) showed that although TEmax ∼200 ms was not equipped to accurately reconstruct either intermediate or/and long T2 pools, it did not have appreciable effects on MWF quantifications. However, a TEmax < 200 ms may not be sufficient to account for signals derived from brain structures with considerably longer T2 components, which may be considered as a limitation of the used GRASE protocol.

## Conclusion

Myelin water fraction measures derived from 3D non-selective GRASE data using the recently proposed reconstruction algorithm have shown to be sensitive for age-dependent alterations of myelin status in the WM of normal-aging brains. The association between mean MWF and RD estimates indicates substantial alterations of myelin content with age throughout several WM regions. Age-related changes of MWFs reflect the physiological evolution of WM myelination between young adulthood to mid-age, followed by a shift toward notable demyelination with higher life-decades. Our findings may valuably contribute to a non-invasive *in vivo* assessment of myelin status in normal-aging human brains.

## Data Availability Statement

All data generated or analyzed during this study are included in this published article (and its Supplementary Information).

## Ethics Statement

The studies involving human participants were reviewed and approved by the local research Ethical Committee Hamburg (Ethik-Komission der Ärztekammer Hamburg) following the guidelines of the Declaration of Helsinki. The patients/participants provided their written informed consent to participate in this study.

## Author Contributions

TF, DK, UH, CT, GB, and JF: conception and design of the study. TF, UH, DK, GB, JN, FF, HL, and CT: acquisition of data. TF, UH, DK, JF, HK, GB, J-PS, FF, HL, RR, GS, AK, CT, and JH: analysis and interpretation of data. All of the listed authors: drafting the article or revising it critically for important intellectual content, final approval of the version to be published, and agreement to be accountable for all aspects of the work in ensuring that questions related to the accuracy or integrity of any part of the work are appropriately investigated and resolved.

## Conflict of Interest

The authors declare that the research was conducted in the absence of any commercial or financial relationships that could be construed as a potential conflict of interest.

## Footnotes

1 https://github.com/muschellij2/extrantsr
