# Supplementary material for: The Myelin Water Fraction Serves as a Marker for Age-Related Myelin Alterations in the Cerebral White Matter – A Multiparametric MRI Aging Study
Source: Front Neurosci. 2020 Feb 24;14:136. doi: 10.3389/fnins.2020.00136 (PMC7050496; doi:10.3389/fnins.2020.00136)
Supplement: Supplementary file 1 [file Table_1.docx]

**Supplementary Information**

The Myelin Water Fraction serves as a Marker for age-related Myelin Alterations in the cerebral White Matter – A Multiparametric MRI Aging Study

**Supplementary Table**

Tables below display the results of multiparametric regression analysis in different white matter regions. The dependent variable is always Myelin Water Fraction (MWF). First column is indicating the assessed region and the independent variable of the model, second, middle and second column from the right are displaying the regression coefficient with the correspondent confidence interval (2.5% and 97.5% respectively). Right column is indicating the p-values for the model. Parameter: RD = Radial Diffusivity; FA = Fractional Anisotropy; MD = Mean Diffusivity; MTR = Magnetization Transfer Ratio.

Regions: SCC = Splenium of corpus callosum; GCC = genu of corpus callosum; CST = corticospinal tract; WM = white matter.

**Frontal WM**

| Region of Interest | Regression Coefficient | 2.5 % | 97.5 % | p-value |
| --- | --- | --- | --- | --- |
| (Intercept) | 0.424 | -0.125 | 0.723 | 0.006 |
| Age (10 years) | -0.008 | -0.012 | -0.003 | <0.001 |
| Frontal RD | -0.038 | -0.053 | -0.023 | <0.001 |
| Frontal FA | 0.011 | -0.007 | 0.029 | 0.221 |
| Frontal MD | -0.000 | -0.005 | 0.005 | 0.940 |
| Frontal MTR | 0.010 | -0.047 | 0.027 | 0.591 |

**Parietal WM**

| Region of Interest | Regression Coefficient | 2.5 % | 97.5 % | p-value |
| --- | --- | --- | --- | --- |
| (Intercept) | 0.416 | 0.197 | 0.635 | <0.001 |
| Age (10 years) | -0.005 | -0.009 | -0.001 | 0.017 |
| Parietal RD | 0.036 | -0.052 | -0.021 | <0.001 |
| Parietal FA | 0.005 | -0.01 | 0.021 | 0.514 |
| Parietal MD | -0.004 | -0.010 | 0.001 | 0.118 |
| Parietal MTR | -0.002 | -0.024 | 0.019 | 0.837 |

**Occipital WM**

| Region of Interest | Regression Coefficient | 2.5 % | 97.5 % | p-value |
| --- | --- | --- | --- | --- |
| (Intercept) | 0.535 | 0.383 | 0.687 | <0.001 |
| Age (10 years) | -0.001 | -0.002 | -0.0 | 0.02 |
| Occipital RD | -0.069 | -0.084 | -0.054 | <0.001 |
| Occipital FA | 0.002 | -0.003 | 0.006 | 0.419 |
| Occipital MD | -0.001 | -0.006 | 0.004 | 0.696 |
| Occipital MTR | 0.003 | -0.010 | 0.016 | 0.633 |

**CST**

| Region of Interest | Regression Coefficient | 2.5 % | 97.5 % | p-value |
| --- | --- | --- | --- | --- |
| (Intercept) | 0.428 | -0.002 | 0.005 | 0.051 |
| Age (10 years) | 0.001 | -0.002 | 0.005 | 0.516 |
| CST RD | -0.076 | -0.092 | -0.059 | <0.001 |
| CST FA | 0.001 | -0.011 | 0.013 | 0.86 |
| CST MD | 0.013 | 0.001 | 0.024 | 0.034 |
| CST MTR | 0.014 | -0.038 | 0.066 | 0.585 |

**SCC**

| Region of Interest | Regression Coefficient | 2.5 % | 97.5 % | p-value |
| --- | --- | --- | --- | --- |
| (Intercept) | 0.043 | -0.292 | 0.378 | 0.797 |
| Age (10 years) | 0.002 | -0.003 | 0.006 | 0.444 |
| SCC RD | -0.072 | -0.095 | -0.048 | <0.001 |
| SCC FA | 0.006 | -0.012 | 0.024 | 0.512 |
| SCC MD | -0.006 | -0.019 | 0.007 | 0.343 |
| SCC MTR | 0.057 | 0.020 | 0.093 | 0.003 |

**GCC**

| Region of Interest | Regression Coefficient | 2.5 % | 97.5 % | p-value |
| --- | --- | --- | --- | --- |
| (Intercept) | 0.274 | 0.093 | 0.455 | 0.004 |
| Age (10 years) | 0.000 | -0.002 | 0.003 | 0.789 |
| GCC RD | -0.052 | -0.068 | -0.036 | <0.001 |
| GCC FA | 0.007 | 0.001 | 0.012 | 0.014 |
| GCC MD | 0.002 | -0.004 | 0.008 | 0.491 |
| GCC MTR | 0.01 | -0.009 | 0.03 | <0.001 |

**Supplementary Figure**

**Distribution of the acquired MRI parameters’ data points throughout the age decades**

1. **Distribution of MWF measures over the age decades**

**
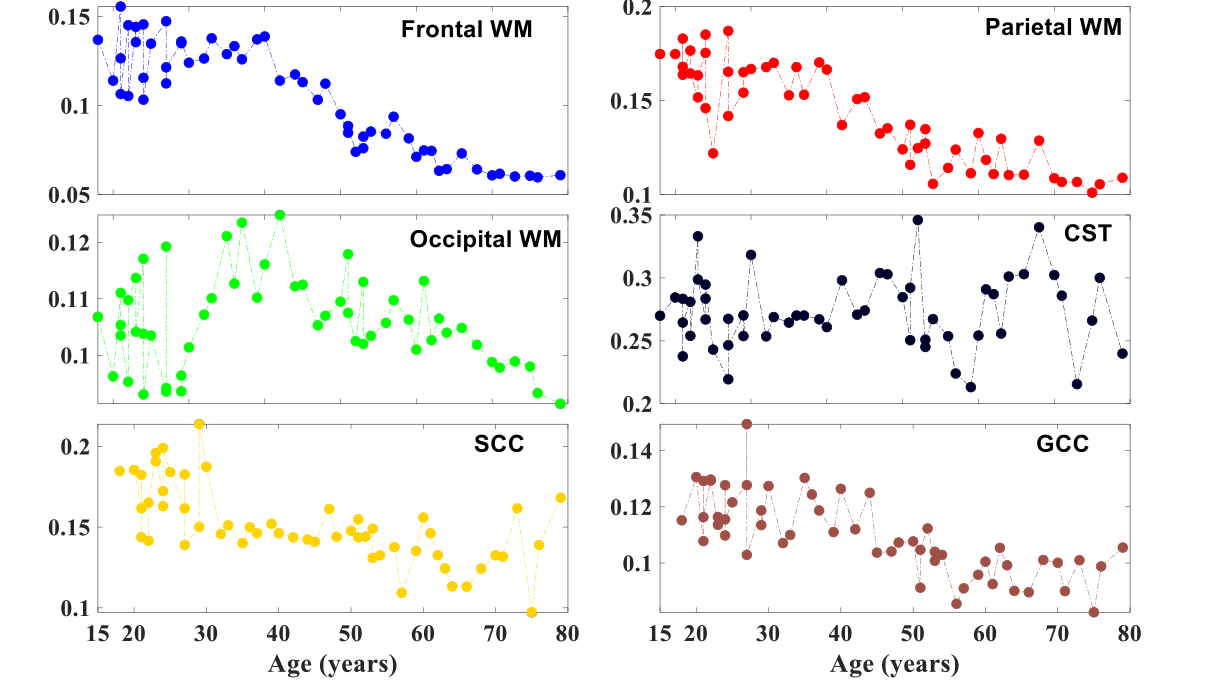
**

1. **Distribution of FA measures over the age decades**

**
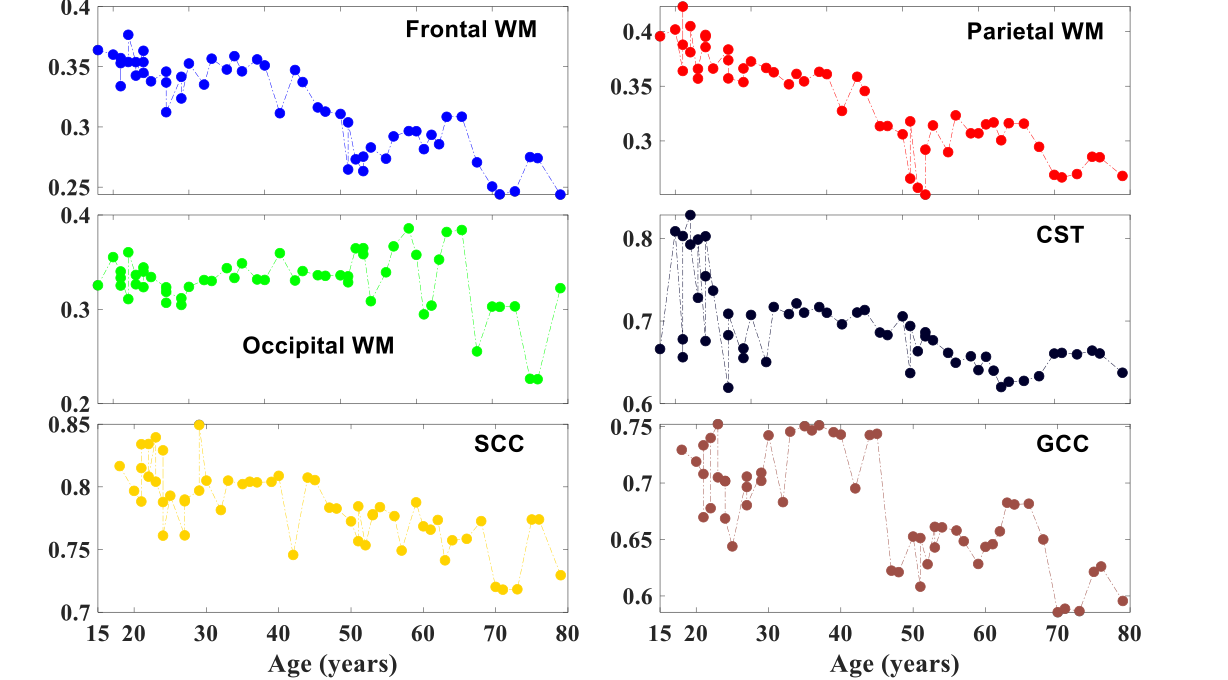
**

1. **Distribution of MD (x10^-3^ mm^2^ /s) measures over the age decades**


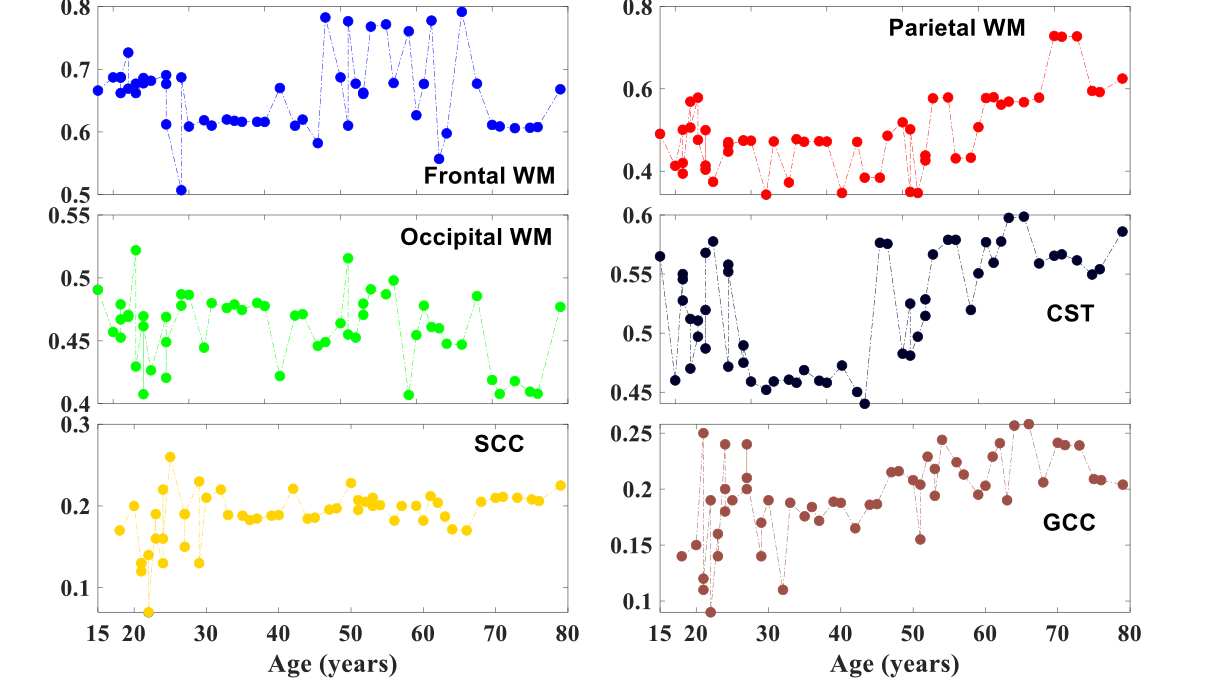


1. **Distribution of RD (x10^-3^ mm^2^ /s) measures over the age decades**


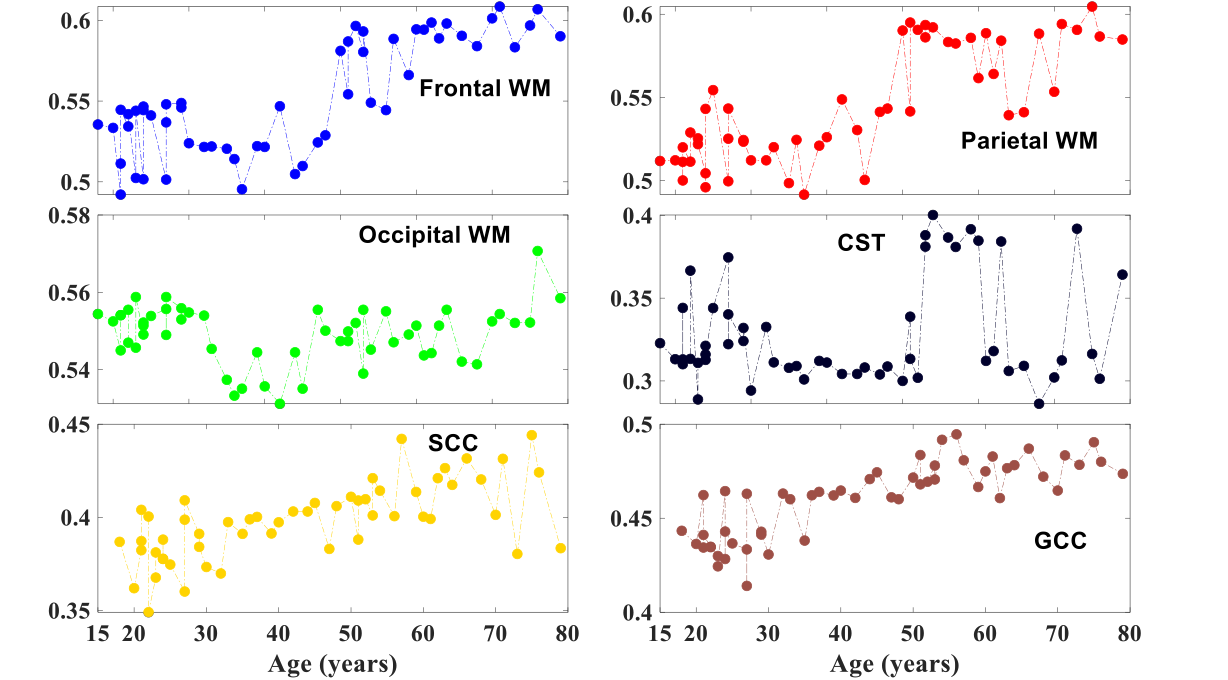


1. **Distribution of MTR measures over the age decades**


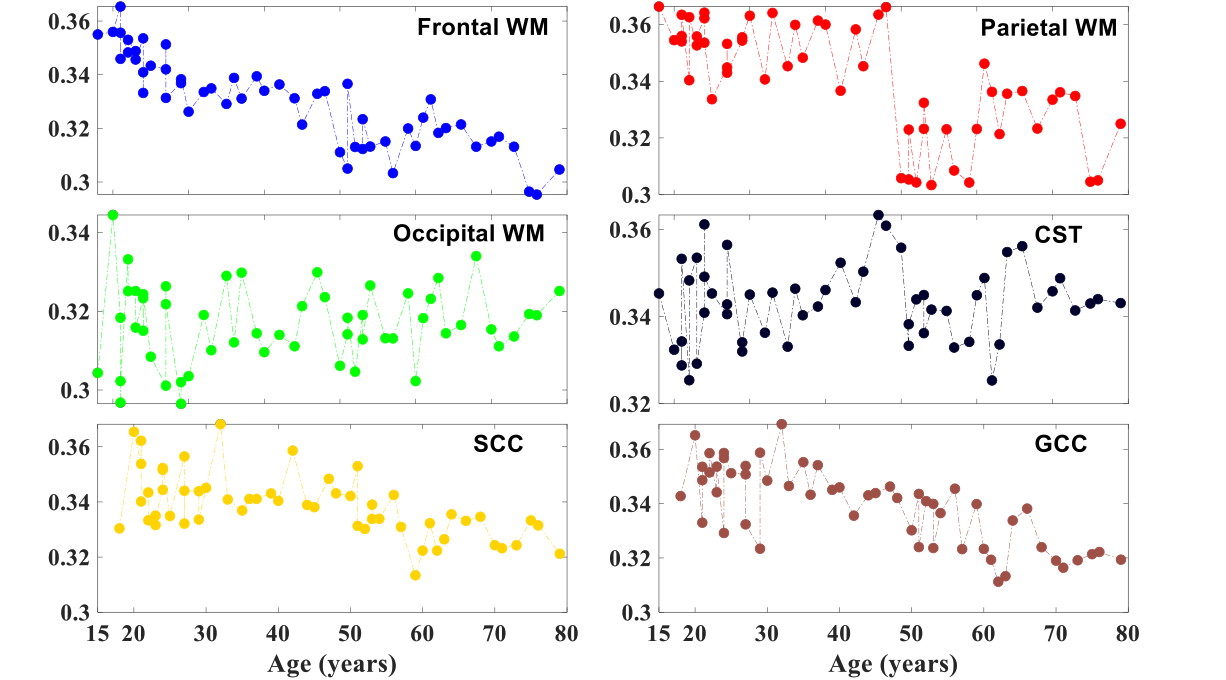


**Legend:** Plots in Supplementary Figure display the exact distribution of each data point derived from each MRI parameter and each patient throughout the observed age decades and brain regions. MWF = Myelin Water Fraction; FA = Fractional Anisotropy; MD = Mean Diffusivity; RD = Radial Diffusivity; MTR = Magnetization Transfer Ratio. **Regions:** White Matter = WM; ROI = region of interest; genu of corpus callosum = GCC; splenium of the corpus callosum =SCC; posterior limb of the corticospinal tract = CST. To improve comparability between the measures and adjust figure scales, MD and RD values are displayed multiplied with the factor 10^3^.
